# Is ^99m^Tc-MIBI scintigraphy a predictor of response to pre-operative neoadjuvant chemotherapy in Osteosarcoma?

**Published:** 2013

**Authors:** Mohammad Gharehdaghi, Vahid Reza Dabbagh Kakhki, Alireza Khooei, Gholamhosein Novferesti, Alireza Hootkani, Mahdi Farzadnia, Ramin Sadeghi

**Affiliations:** 1 Department of Orthopedic surgery, Imam Reza Hospital, Mashhad University of Medical Sciences, Mashhad, Iran; 2 Nuclear Medicine Research Center, Ghaem Hospital, Mashhad University of Medical Sciences, Mashhad, Iran; 3 Department of Pathology, Imam Reza Hospital, Mashhad University of Medical Sciences, Mashhad, Iran; 4 Department of Oncology, Omid Hospital, Mashhad University of Medical Sciences, Mashhad, Iran

**Keywords:** Osteosarcoma, ^99m^Tc-MIBI, Therapy response, Neoadjuvant chemotherapy

## Abstract

**Objectives:**

Multidrug resistance (MDR), which may be due to the over expression of P-glycoprotein (Pgp) and/or MRP, is a major problem in neoadjuvant chemotherapy of osteosarcoma. The aim of this study was to investigate the role of Tc-99m MIBI scan for predicting the response to pre-operative chemotherapy.

**Methods:**

Twenty-five patients (12 males and 13 females, aged between 8 and 52y) with osteosarcoma were studied. Before the chemotherapy, planar ^99m^Tc-MIBI anterior and posterior images were obtained 10-min [tumor-to-background ratio: (T_1_/B_1_)_10min_] and 3-hr after tracer injection. After completion of chemotherapy, again ^99m^Tc-MIBI scan was performed at 10-min after tracer injection. In addition to calculation of decay corrected tumor to background (T/B) ratios, using the 10-min and 3-hr images of the pre-chemotherapy scintigraphy, percent wash-out rate (WR%) of ^99m^Tc-MIBI was calculated. Using the 10-min images of the pre- and post-chemotherapy scans, the percent reduction in uptake at the tumor site after treatment (Red%) was also calculated. Then after surgical resection, tumor response was assessed by percentage of necrosis.

**Results:**

All patients showed significant ^99m^Tc-MIBI uptake in early images. Only 9 patients showed good response to chemotherapy (necrosis≥90%) while 16 patients were considered as non-responder (necrosis<90%). There was no statistical significant difference between non-responders and responders in (T_1_/B_1_)_10min_.There was a significant negative correlation between WR% and percentage of necrosis (P=0.001). On the other hand, there was a significant correlation between Red% and percentage of necrosis (P<0.001).There was also statistical significant difference in WR% and Red% between non-responders and responders (both P< 0.001).

**Conclusion:**

Washout rate of ^99m^Tc-MIBI in pre-chemotherapy scintigraphy as well as Red% using pre- and post-chemotherapy MIBI scintigraphy are useful methods for predicting response to neoadjuvant chemotherapy.

## Introduction

Osteosarcoma is the most frequent primary malignant bone tumor and occurs most frequently in children and adolescents (1, 2). In bone and soft-tissue malignant tumors, preoperative neoadjuvant chemotherapy has been a standard therapeutic method (3). The histological response to pre-operative neoadjuvant chemotherapy is a reliable predictive parameter for survival in osteosarcoma (2). However, multi drug resistance (MDR) is a major problem which includes over expression of drug resistance proteins, such as MDR1 P-glycoprotein (Pgp), and MDR associated protein (MRP) (3, 4). It is reported that MDR1 expression correlates with resistance to chemotherapy in osteosarcoma (4-6). Some chemotherapic agents are substrates of Pgp and MRP, including doxorubicin, which is the most effective agent for osteosarcoma management (1). Pgp acts as an adenosine triphosphate-dependent efflux pump to reduce the intracellular accumulation of chemothe- rapeutic agents (4).

^99m^Tc-MIBI is a promising tumor-imaging agent including bone and soft-tissue tumors (2, 7). Some studies showed that ^99m^Tc-MIBI to be a substrate for both Pgp and MRP (7-10). A number of studies have reported that the early ^99m^Tc-MIBI uptake is inversely correlated with Pgp levels (7,9,11-13) as well as in patients with musculoskeletal sarcomas significant correlation has been reported between the efflux rate of ^99m^Tc-MIBI and the Pgp level expression (3, 9).

In this study, ^99m^Tc-MIBI scintigraphy in patients with newly diagnosed osteosarcoma was evaluated. For this purpose, we compared ^99m^Tc-MIBI uptake (before and after neoadjuvant chemotherapy) as well as ^99m^Tc-MIBI washout rate with the response to pre-operative neoadjuvant chemotherapy determined by level of necrosis on histopathological examination.

## Methods

### 

#### Patients

Twenty five consecutive (12 males, 13 females) patients with a diagnosis of osteosarcoma in one extremity were studied. Their age was 20.48±9.60 y (range, 8-52 y). Before starting any treatment including neoadjuvant chemotherapy all patients underwent ^99m^Tc-MIBI scintigraphy. After that the patients received Cisplatin and Adriamaycin as preoperative neoadjuvant chemotherapy. Before the surgical resection (after completion of chemotherapy) ^99m^Tc-MIBI scintigraphy was repeated. The local ethics committee approved the study protocol; and informed consent was taken from the all patients or their parents prior to the study.

#### ^99m^Tc-MIBI Scintigraphy

All patients underwent two examinations using ^99m^Tc-MIBI: before commencing neoadjuvant chemotherapy and after completing all preoperative chemotherapy cycles. 10-min and 3-h after IV injection of 600-740 MBq ^99m^Tc-MIBI, whole body anterior and posterior planar images as well as spot images were obtained. Images were acquired using a Dual-head gamma-camera (Dual-Head Variable-Angle E.CAM; Siemens) equipped with low energy, high-resolution parallel hole collimator. Energy photo-peak was set at 140 keV with a 20% symmetric window.

Images were evaluated visually and semi-quantitatively. A manual region of interest (ROI) was drawn around the lesion (T) and an identical mirrored ROI was placed on the contralateral limb (B). Tumor-to-background (T/B) ratios were determined from the mean counts in early (10-min) and delayed images (3-h) acquired before the neoadjuvant chemotherapy as (T_1_/B_1_)_10min_ and (T_1_/B_1_)_3hr_ respectively. After decay correction, the percent washout rate (WR%) of ^99m^Tc-MIBI from the tumor was determined using the following formula:

WR% = [[(T_1_/B_1_)_10min_-(T_1_/B_1_)_3hr_]/(T_1_/B_1_)_10min_] ×100

Again after neoadjuvant chemotherapy, all patients were re-scanned using ^99m^Tc-MIBI imaging and planar images were obtained at 10-min after tracer injection.

Percent reduction (Red%) in tumor uptake was estimated by comparing the activities at the tumor site before and after the therapy. Taking the counts from pre-chemotherapy [(T_1_/B_1_)_10min_] and post-chemotherapy [(T_2_/B_2_)_10min_] scans; following formula was used:

Red% = 100 × [(T_1_/B_1_)_10min_- (T_2_/B_2_)_10min_]/(T_1_/B_1_)_10min_

After surgical resection of tumor, histopatholo- gic examination was used for assessment of response to preoperative neoadjuvant chemotherapy. Based on Huvos grading system, percentage of histological necrosis was categorized in four groups: grades: I<50%, 50%≤II<90%,: 90%≤ III ≤99%, and IV:100% necrosis respectively. Based on this grading system, poor response was described as: Grades I and II, while grades III and IV indicate a good response. Patients with a percentage of necrosis of ≥90% (good response) were considered as responders at all.

#### Statistics

T/B ratios, WR% and Red% of ^99m^Tc-MIBI uptake were compared with therapy response. Values are presented as mean ±SD. Two-tailed unpaired Student’s t-test; Pearson correlation coefficient and simple regression were used for comparison. Comparison between categorical variables was performed using ANOVA in post-Hoc test analysis (Tukey’s test). The significant level was considered as *p*<0.05.

## Results

Locations of osteosarcomas were: 13 in the distal femur, 6 in the proximal tibia, 3 in the distal tibia, 1 in the proximal humerus and 2 in the distal radius.

All patients visually showed significant uptake in the tumor site at the 10-min image of pre-chemotherapy ^99m^Tc-MIBI scan. Mean±SD of the (T_1_/B_1_)_10min,_ (T_1_/B_1_)_3hr_, (T_2_/B_2_)_10min_, WR%, Red% and percentage of histological necrosis were 3.47±1.60, 1.96±0.77, 2.20±1.10, 39.81%±13.81, 43.56%±18.17 and 76.44%±19.40 respectively. Nine patients showed good response to chemotherapy (necrosis≥90%) while 16 patients were considered as non-responder (necrosis<90%).

### 

#### Early uptake ratio

There was a significant correlation between early tumor to background uptake ratio [(T_1_/B_1_)_10min_] on pre-chemotherapy scan and percentage of necrosis(r = 0.595 and *P*=0.02). However, there was no statistical significant difference between non-responders and responders in (T_1_/B_1_)_10min_ (3.77±1.6 and 2.94±1.6 respectively, *P*= 0.2). On the other hand one way ANOVA showed no significant difference in (T_1_/B_1_)_10min_ between four categorized groups based of percentage of necrosis.

#### Washout Rate (WR%)

**Figure 1:**
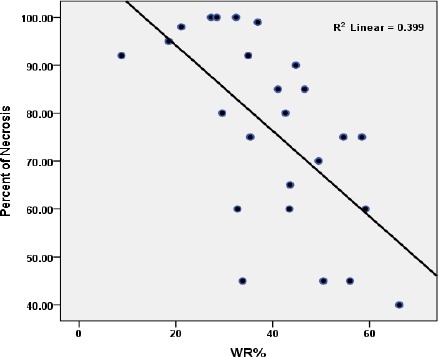
Linear regression between WR% and percent of necrosis.

There was a significant correlation between WR% and percentage of necrosis (r= -0.632 and *P*=0.001). There was also statistical significant difference between non-responders and responders (WR%=46.41±10.6 and WR%=28.09±10.8 respectively, *P*<0.001). Although ANOVA showed significant difference in WR% between four groups based of response grading (*P*=0.008), but in Post Hoc Test analysis there was only significant difference between patients with grade II and III (*P*=0.013). Linear regression analysis between WR% and percentage of necrosis is shown in Figure 1.

#### Percentage reduction of tumor uptake (Red%)

**Figure 2:**
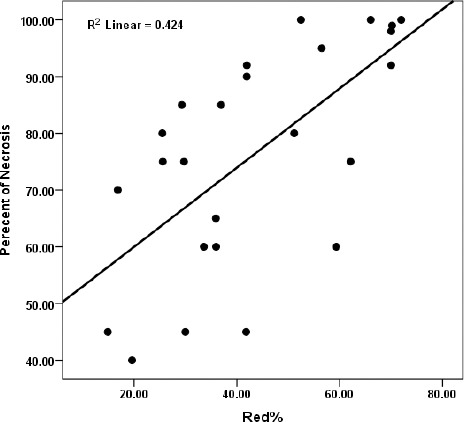
Linear regression between Red% and percent of necrosis.

There was a statistically significant but modest correlation between Red% and percentage of necrosis (r = 0.651 and *P*<0.001). There was also statistical significant difference between non-responders and responders (Red% =34.25±13.82 and Red% =60.10±12.78 respectively, *P*< 0.001). Although ANOVA showed significant difference in Red% between four categorized groups (*P*=0.002) but on Post Hoc Tests, there was significant difference between 4 histological grades except between grade I and II as well as III and IV. Figure 2 shows the linear regression between Red% and percentage of necrosis.

#### WR% and Red%

There was a significant correlation between WR% and Red% (r= -0.727 and *P*<0.001).

## Discussion

The main cause of treatment failure in osteosarcoma is MDR, so preoperative risk evaluation to predict response to chemotherapy is important. Pgp and MRP expression were reported to be associated with drug resistance, recurrence, and poor prognosis in patients with osteosarcoma (1, 14, 15). In vitro studies showed that ^99m^Tc-MIBI is a substrate of both Pgp and MRP efflux pumps (7-10, 16). It was basic concept for clinical investigations in various tumors using ^99m^Tc-MIBI (1-4, 11-13, 16-18).

In our series, response to preoperative neoadjuvant chemotherapy was determined by percentage of necrosis in the histopathological examination. Based on ≥90% tumor necrosis as responders, 16 of 25 patients were non-responders. In this study, there was inverse correlation between WR% of ^99m^Tc-MIBI and percentage of necrosis and it was higher in non-responders. On the other hand, we did not find significant difference in early T/B uptake ratio [(T_1_/B_1_)_10min_] of pre-chemotherapy ^99m^Tc- MIBI scan between responders and on-responders (based on percentage of necrosis ≥ 90%). Lower early T/B ratio in tumors with significant necrosis in presentation time may be due to poor vascularization (9).

Burak *et al* (9) and Taki *et al* (3), observed that the WR% of ^99m^Tc-MIBI in musculoskeletal sarcomas was correlated with the degree of Pgp expression. Burak *et al* (9) also didn’t find a significant correlation between tumor to background uptake ratio of ^99m^Tc-MIBI and Pgp expression. They also didn’t observe relationship between the level of expression of Pgp and the extent of tumor necrosis, in contrast to the findings of Baldini *et al* (15). The difference between T/B ratios of patients with high and patients with low Pgp expression was also not significant (9). Sohaib *et al* studied 31 patients with bone and soft tissue sarcoma. They found that tumor to background ratio correlated poorly with the tumor necrosis values in the specimen (R=0.23 and 0.06 respectively). There was weak correlation between tumor necrosis and WR% (r=-0.32, *P*=0.029) (19).

In our study, we can conclude indirectly that WR% of ^99m^Tc-MIBI seems to be a good indicator of the efflux pump functions. On the other hand, Red% index in our study showed correlation with response to neoadjuvant chemotherapy (percentage of necrosis). This finding may indicate that the degree of ^99m^Tc-MIBI uptake reflects some tumor metabolism. It should be considered that by WR% we can predict the therapy response before installing chemotherapy.

Although our findings are similar to some other studies about ^99m^Tc-MIBI scintigraphy in osteosarcoma, we have to mention some reports are not compatible with our findings. There are still some points that need to be evaluated and clarified. The expression of transmembrane transporter proteins is not enough to define the MDR in osteosarcoma (1). On the other hand, the functional transport capacity of these drug efflux pumps should be considered. Indeed, there is an uncoupling between Pgp expression and the level of its action (1, 9). Many studies reported that the overexpression of Pgp is a significant indicator in prediction of response to neoadjuvant chemotherapy, whereas others report that the level of Pgp expression is not correlated with therapy failure (1, 9, 21).

Methodological differences can described some controversies between different studies (1). Although there is significant overlap between Pgp and MRP in substrate specificity, some major differences have been reported (1). MRP may act as a glutathione-S-conjugate efflux pump (GS-X pump) and over expression of glutathione-S-transferase may have an effect on failure of preoperative chemotherapy (22). So further studies with large number of patients are needed to clarify the interaction between MRP and GSH in osteosarcoma. The heterogeneous distribution of transmembrane transporter proteins in osteosarcoma may have an effect for different results (9). We should considered *in vivo* complexity in uptake, washout and kinetic of ^99m^Tc-MIBI (9). In addition to the MDR-related proteins, ^99m^Tc-MIBI kinetic depends on various biological factors, such as blood flow, capillary permeability, necrotic and stromal components of the tumor(9). Several factors may have an influence in diminished ^99m^Tc-MIBI uptake in the tumor: Poor vascularization and accessibility of ^99m^Tc-MIBI to the tumor, early stage of apoptosis, decreased viability and electrical gradients in “over-aged” and hypoxic cells, multidrug resistance proteins and/or over expression of the anti-apoptotic protein Bcl-2, preventing any mitochondrial accumulation (23). Distinguish between apoptotic cells and resistant cells may be impossible using ^99m^Tc-MIBI alone since this tracer is not accumulated in either of these cell types (23).

The accuracy of our findings should be reproduced by further studies with an extended number of patients and long follow-up to clarify the different mechanisms in efflux of ^99m^Tc-MIBI from malignant cells, non-MDR-related mechanisms, and prognostic implication of ^99m^Tc-MIBI.

## Conclusion

- High washout rate of ^99m^Tc-MIBI is probably useful method for predicting of the response to neoadjuvant chemotherapy;
- Uptake level of ^99m^Tc-MIBI in pre-chemotherapy scan may not be useful, so wash-out analysis of ^99m^Tc-MIBI using early and delayed images is recommended to predict the response;
- Reduction in ^99m^Tc-MIBI uptake using pre- and post-chemotherapy ^99m^Tc-MIBI scan is useful for assessment of response to chemotherapy;
- Regarding the controversial findings in our study and previous investigations, further studies especially assessment of prognosis are recommended.
